# Type 1 Endoleak: Management following Thoracic Endovascular Aortic Repair

**DOI:** 10.1055/s-0042-1756665

**Published:** 2022-12-15

**Authors:** Martin Czerny, Maximilian Kreibich, Tim Berger, Stoyan Kondov, Matthias Siepe, Friedhelm Beyersdorf, Bartosz Rylski

**Affiliations:** 1 Department of Cardiovascular Surgery, University Hospital Freiburg-Heart Center, Freiburg, Germany; 2 Faculty of Medicine, Albert-Ludwigs-University, Freiburg, Germany

**Keywords:** TEVAR, type IA endoleak, FET

## Abstract

The best treatment option for type IA endoleak after thoracic endovascular aortic repair (TEVAR) is its avoidance by understanding the underlying disease process, having/creating adequate landing zones, as well as respecting anatomy in combination with knowledge of the capabilities and limitations of the TEVAR device used.

## Introduction

**Fig. 1:**
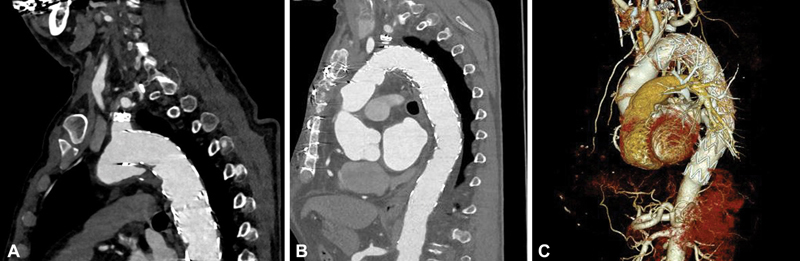
Representative computed tomography angiography of a type IA endoleak ( **A** ) following zone 2 thoracic endovascular aortic repair, subclavian-to-carotid bypass, and proximal subclavian artery occlusion to treat an acute, complicated Type B aortic dissection. The asymptomatic endoleak was diagnosed during a routine follow-up visit in our dedicated aortic clinic 4 years following the procedure. The patient was treated by frozen elephant trunk implantation thereafter. Postoperatively, the endoleak was successfully excluded ( **B, C** ) and the patient was discharged home.

Type I endoleaks following thoracic endovascular aortic repair (TEVAR) do occur ( Fig. 1A ). This may be due to the progression of the underlying disease process, but it may also reflect failed planning for the index procedure, occurring early or late, and usually associated with inadequate landing zones, with regard to shape or length. 1 2 3 The scope of this article is to provide the interested reader with a short cookbook on how to avoid endoleak occurrence and, in case of being confronted with the situation, which treatment options to choose in different clinical scenarios.

## Underlying Mechanisms and Extent of Disease

The success or failure of TEVAR with regard to proximal sealing is to a very large extent dependent on the length of the proximal landing zone as well as its shape. This per se is dependent on the correct interpretation of the underlying pathology. Here, a differentiation has to be made between dilatative and obliterative arteriopathies. Obliterative ones are slightly more forgiving, as medial sclerosis is usually extensive, so dilatation of the proximal landing zone occurs very rarely. The situation is different in dilatative arteriopathy, irrespective of classical aneurysm or postdissection aneurysmal formation. In both, a tendency to progression of the underlying disease process is present. Even more importantly, the length of the proximal landing zone cannot be overemphasized. The proximal landing zone should have a length of either healthy or at least morphologically adequate aorta of at least 2.0 cm (ideally >2.5 cm). 4 5 6 In addition, the aortic arch configuration has to be taken into account, as the majority of patients with descending thoracic aortic pathology involving the distal aortic arch are type III arches, which translates into the need to have a long zone of alignment in the arch per se. Otherwise, an angulation of more than 90 degree follows which impairs the stability even if the length of the landing zone is adequate. 7

## Conceptual Treatment Approaches and Options

The aim of each approach is to achieve permanent exclusion of the lesion from the bloodstream irrespective of pathology, by preventing type I endoleak formation. Four main options are available, along with a few others, usually representing trade-offs.

**Fig. 2:**
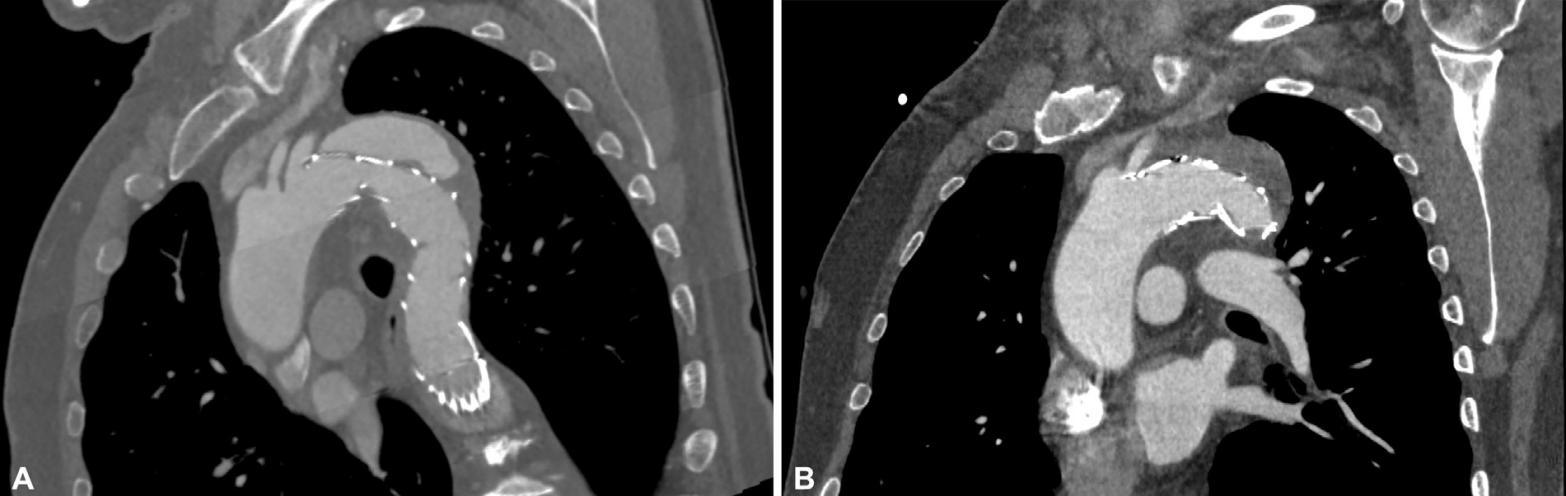
Representative computed tomography angiography of a type IA endoleak ( **A** ) following zone 3 thoracic endovascular aortic repair (TEVAR) to treat an acute complicated Type B aortic dissection. ( **B** ) The endoleak was visible in the postoperative control, and the patient was treated shortly thereafter with a subclavian-to-carotid bypass and proximal TEVAR extension into zone 2.

1. *Extension of the proximal landing zone by transposition of the supra-aortic branches* : transposition of the supra-aortic branches was established several years ago as a highly effective and durable treatment option for landing zone extension. 3 8 While the results of subclavian-to-carotid transposition/bypass, as well as the results of double transposition, have been excellent, total arch rerouting has been largely abandoned due to the high rate of retrograde Type A aortic dissection, in particular in patients with the underlying diagnosis of Type B aortic dissection. Having suffered Type B aortic dissection indicates an inherent aortic disease also in morphologically normal segments. 9 These approaches per se can also be used for secondary landing zone extension in case of type IA endoleak formation ( Fig. 2A,B ), when the desired landing zone length of 2.0 cm (ideally, 2.5 cm) can be achieved, in cases where the neck shape and the angulation to the descending aorta are adequate.
2. *Frozen elephant trunk (FET) implantation (open surgical approach)* : the FET technique has been established for the treatment of several acute and chronic thoracic aortic pathologies involving the aortic arch. Mid-term results are excellent. 10 11 The FET technique also represents an excellent means for the treatment of Type IA endoleak formation, as it presents what we call a “proximal full fix,” eliminating all native tissue and, thereby, preventing any potential recurrence of disease ( Fig. 1B,C ). As many affected endoleak patients will also require some form of treatment of either structural heart valve disease and/or coronary artery disease, this approach fixes the entire issue.
3. *Collar-based solutions (open surgical approach)* : in several scenarios, “a disease has been implanted into a disease” during the index procedure, meaning that a 46-mm or even 48-mm stent-graft has been implanted in an ectatic proximal landing zone. This mistaken approach even enhances the process of proximal dilatation. As the largest stent-graft components of the currently available FET prostheses have smaller diameters, an add-on is needed. A collar-based solution such as the Siena prosthesis from Terumo Aortic may be one option. The large sewing collar of the Sienna graft adapts individually to any proximal stent-graft diameter, thereby enabling very flexible tailoring of the individually needed solution. 12
4. *Branched endovascular aortic arch repair* : branched endovascular aortic arch repair has been established as an alternative to classical aortic arch replacement, and its clinical use is rising. However, several anatomical conditions need to be present to enable its safe and effective use. In case double transposition is not able to create a sufficient proximal landing zone and in case the FET technique is deemed not suitable, the branched endovascular aortic arch approach is an excellent option to treat type IA endoleak formation. 13

### Additional (Less Appealing) Approaches: Coiling/Glueing/Endoanchoring

Application of glue and coils may induce thrombosis and create a nice postoperative computerized tomography scan, but it remains speculative if these approaches will also decompress the lesion. Therefore, they should be considered as options but used with reluctance. Finally, endoanchoring is technically feasible but should also be seen as a last resort and not as a first option, due to the known limitations. 14

Parallel grafts: parallel grafts, due to their inefficacy, have also been largely abandoned and should only be used in acute clinical scenarios where no other options are available. 4

## Conclusion

In conclusion, avoidance remains the best option for type IA endoleak. If all aforementioned recommendations of avoidance are applied, the remaining risk of type IA endoleak occurrence can be reduced to a minimum. Nevertheless, in case of endoleak occurrence, availability of the entire spectrum of options—extension of the proximal landing zone by supraaortic transposition, FET implantation, use of the Siena prosthesis, and finally branched endovascular aortic arch repair should all be available under one umbrella to enable the right treatment choice in the right clinical scenario in the right patient.
